# Revealing the Intrinsic Correlation between Cu Scales and Free Radical Chain Reactions in the Regulation of Catalytic Behaviour

**DOI:** 10.3390/molecules29194690

**Published:** 2024-10-03

**Authors:** Haifeng Zhang, Zilong Zhang, Jingyi Yan, Siyang Wang, Xubin Huang, Fangmin Zuo, Ao Li, Fengkai Gao, Haidan Lin, Bolin Wang

**Affiliations:** 1 School of Chemical Engineering, Northeast Electric Power University, Jilin 132012, China; 2 Tianjin Key Laboratory of Molecular Optoelectronic Science, Department of Chemistry, School of Science, Tianjin University, Tianjin 300072, China; 3 Science and Technology Industry Division, Northeast Electric Power University, Jilin 132012, China; 4 Electric Power Research Institute, State Grid Jilin Electric Power Co., Ltd., Changchun 130012, China

**Keywords:** copper-based catalyst, oil-paper insulation, active site, free radical reaction, DBPC

## Abstract

Defining the copper-based catalysts that are responsible for the catalytic behaviour of oil-paper insulation systems and implementing effective regulation are of great significance. Accelerated ageing experiments were conducted to reveal variations in copper scales and deterioration in insulation properties. As ageing progressed, TEM images demonstrated that copper species were adsorbed and aggregated on the fibre surface in the form of nanoparticles (NPs). The scale of NPs exhibited a continuous increase, from 27.06 nm to 94.19 nm. Cu(I) and Cu(II) species were identified as the active sites for inducing intense free radical reactions, which significantly reduced the activation energy, making the insulating oil more susceptible to oxidation. The role of the antioxidant di-tert-butyl-p-cresol (DBPC) in extending the insulation life was regulated by determining the optimal addition time based on variations in the interfacial tension. After the second addition of DBPC, the ageing rates of the dissipation factor, acidity, micro-water and breakdown voltage in the Cu+DBPC group decreased by 28.8%, 43.2%, 52.9% and 46.7%, respectively, compared to the Cu group. This finding not only demonstrates the crucial role of DBPC in preventing the copper-based catalyst-induced oxidation of insulating oil, but also furnishes a vital foundation for enhancing the long-term stability of transformer insulation systems.

## 1. Introduction

Power transformers are important hub equipment to ensure the safe and efficient transmission and use of electricity [1,2]. The optimisation of voltage and transmission capacity is of particular importance in light of the advancements being witnessed in modern power systems [3]. The lifespan of an oil-immersed transformer is considerably contingent upon the functionality and deterioration of the oil-paper insulation system [4]. The roles of insulation, cooling, arc extinguishing and carrying information are assumed by insulating oil [5,6,7]. Insulating paper, as the principal solid insulating material, is mainly used to inhibit the current’s flow between conductors and supply certain mechanical supports [8,9]. Moreover, most of the oil-immersed transformers used worldwide are free-breathing transformers. Despite the effectiveness of breathers in controlling humidity, they are ineffective in preventing the ingress of oxygen into a transformer via the breather, which considerably accelerates the degradation of insulation performance [10].

As one of the most hazardous metals, the catalytic oxidation of copper in an insulation system cannot be ignored. To prevent and reduce transformer failures, the mechanisms of dissolved copper formation, insulation oil deterioration and insulation paper degradation have become cutting-edge research for transformer failure warnings [11,12,13,14,15,16,17,18,19,20,21]. In addition, researchers have comprehensively evaluated the ageing characteristics of transformer oil-paper insulation from various perspectives, including the rational design of different types of oil, the modified design of insulating paper, the structural optimisation of copper windings and the mutual adaptability of oil-paper [22,23,24,25,26]. These results reveal the formation mechanism of transformer insulation faults and lay a theoretical foundation for transformer condition assessment, lifetime analysis and fault diagnosis technology. However, monitoring and implementing the effective regulation of the catalytic behaviour of copper-based catalysts in service transformers has been the direction of efforts. As the ageing time progresses, the available antioxidants in the oil are depleted, leading to further deterioration of the insulating properties. The antioxidant performance of insulating oils is constantly improving when the level of antioxidants in the oil is within 0–1%. However, the excessive addition of di-tert-butyl-p-cresol (DBPC) is not essential. On the one hand, the addition of antioxidants ionises hydrogen ions at high temperatures, which is an important factor in the accelerated ageing of insulating paper. On the other hand, adding excessive concentrations of DBPC causes unnecessary economic costs. Therefore, defining the time point of DBPC addition to achieve optimal insulation properties during the ageing process remains a significant challenge.

Inspired by the above principles, the ageing rate of oil-paper insulation at the same time interval was explored at 403 K. Multiple characterisation techniques were used to elucidate the active sites that initiate the catalytic oxidation of the oil-paper insulation system. The DBPC incorporation node was determined by the variation in the interfacial tension. The inclusion of DBPC significantly inhibited the ageing rate of the physicochemical and electrical properties of the oil-paper insulation system, demonstrating that the insulation properties were effectively regulated. This study elucidates the wide range of possibilities offered for technological advances in the early warning of transformer oil failures.

## 2. Result and Discussion

**Figure 1:**
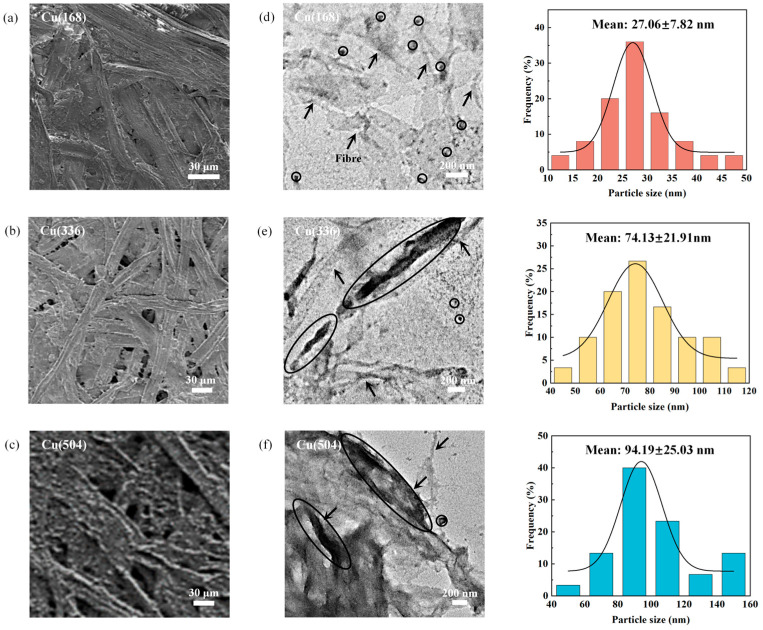
SEM images of (**a**) Cu(168), (**b**) Cu(336) and (**c**) Cu(504); TEM images and particle size distribution of (**d**) Cu(168), (**e**) Cu(336) and (**f**) Cu(504).

The morphology of insulating paper with different ageing times was analysed using scanning electron microscopy (SEM) as shown in Figure 1a–c. IP(168), IP(336), IP(504), Cu(168), Cu(336) and Cu(504) are oils containing only insulating paper and paper-coated copper with the ageing times of 168 h, 336 h and 504 h, respectively (see Supplementary Materials). The presence of flocculated material on fibre surfaces is typically indicative of physical and chemical interactions between the fibres. The formation of a tight network structure between cellulose molecules is facilitated by hydrogen bonding, with the stability of this structure influenced by the number of hydroxyl groups [27]. Copper reacts with the hydroxyl groups in cellulose, thereby reducing their affinity for forming hydrogen bonds. Consequently, the reduction in hydrogen bond strength results in an increase in inter-fibre porosity, rendering the fibres susceptible to pore formation [28,29,30]. Furthermore, the presence of copper initiates specific chemical reactions, such as oxidation, which further compromises the structural integrity of the cellulose, ultimately leading to fibre ageing and degradation [31,32]. This degradation process results in the formation of additional pores and defects, thereby increasing the porosity of the fibres. The distribution and deposition of copper species on the insulating paper surface were further determined by scanning electron microscopy coupled with energy dispersive X-ray spectrometry (SEM-EDS) and inductively coupled plasma-mass spectrometry (ICP-MS), as shown in Figure S1 and Table S1. In addition, the distribution of copper species on the fibre surface was analysed using transmission electron microscopy (TEM), as shown in Figure 1d–f. The circled areas represent the primary copper species, and the arrows point to the fibres. The particle size of the copper, in the form of nanoparticles (NPs), that adhered to the fibre surface exhibited a progressive increase from 27.06 nm to 94.19 nm with ageing time. On the one hand, the copper was dissolved from the copper sheet surface and diffused into the oil and then migrated to insulating paper through the oil-paper interface. The fibres underwent fracture and degradation, which led to an increase in their surface porosity, and these pores provided more cavities for trapping the copper species. On the other hand, the increased surface roughness of the fibres led to an increase in surface charge adsorption and more copper species were adsorbed onto the fibre surface for deposition and aggregation, resulting in a gradual increase in the particle size of the copper species. The adsorbed copper species are described in detail below.

**Figure 2:**
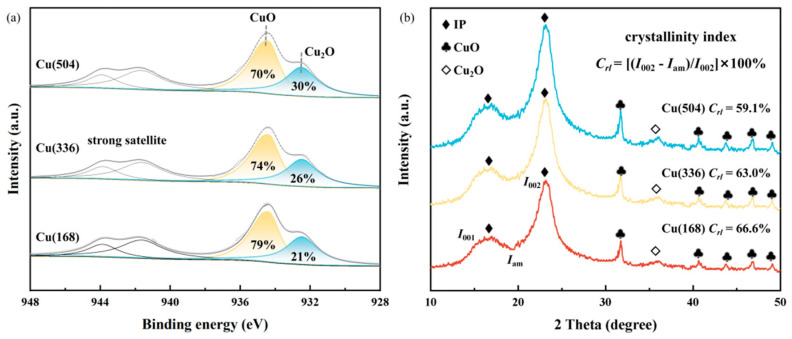
Different ageing times of (**a**) XPS spectra; (**b**) XRD patterns.

There is no denying that copper can be precipitated as oxides, sulphides, or other compounds and complexes, or even as metallic copper following redox processes [33]. To further explore the existential states of the Cu species, X-ray photoelectron spectroscopy (XPS) was performed. As shown in Figure 2a, the peak at 934.7 eV was assigned to CuO, while a strong satellite oscillation peak was identified to confirm the presence of the CuO species [34]. The peak observed at 932.4 eV may be attributed to the presence of either Cu_2_O or Cu(0), given that their respective binding energies exhibit a notable degree of similarity [34]. The Cu LMM Auger electron spectra are shown in Figure S2. Additionally, 918.5 eV was attributed to CuO species and 916.8 eV to Cu_2_O species [35]. Notably, no Cu(0) species were detected. X-ray diffraction (XRD) was used to characterise the extent of the microdestruction of cellulose, where there are regular chains of crystalline regions and disordered amorphous regions. The crystallinity of cellulose is used to describe the percentage of cellulose molecules in the whole crystal structure. Cellulose molecules with a high degree of crystallinity possess remarkably elevated flexibility and are the basis for insulating papers with excellent mechanical strength. As shown in Figure 2b and Figure S3, the crystalline zone (002) and amorphous zone (001) were observed for insulating papers with different ageing times [36,37]. The crystallinity index of the insulating paper exhibited a gradual decline as the ageing time increased. This evidence suggests that the molecular structure of the cellulose was damaged, which is consistent with the results presented by TEM and SEM. The diffraction peaks at 2θ = 32.6°, 40.6°, 43.7°, 46.9° and 49.1° coincide with CuO crystals [38]. Cu_2_O crystals are identified by the distinctive diffraction peaks at 2θ = 36.2° [39]. The Cu_2_O diffraction peaks became progressively stronger with time, probably representing a partial conversion of CuO to Cu_2_O. This is consistent with the XPS results and further confirms that copper is deposited on the fibre surface in the form of oxides. Additionally, the Raman spectra of Cu(504), as shown in Figure S4, revealed typical peaks of 217, 298, 346 and 632 cm^−1^, proving the presence of a mixed phase of Cu_2_O and CuO [40,41].

**Figure 3:**
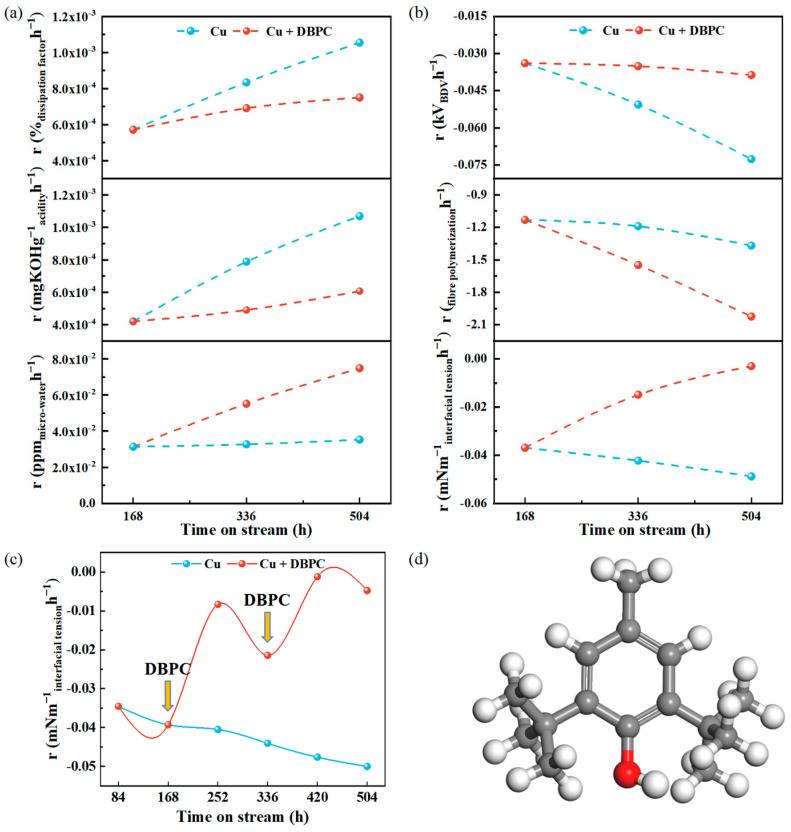
(**a**) Insulation properties (dissipation factor, acidity and micro-water) with different ageing times; (**b**) insulation properties (breakdown voltage, fibre polymerisation and interfacial tension) with different ageing times; (**c**) trend in interfacial tension with DBPC addition; (**d**) DBPC structure. The white, grey and red spheres represent the H, C and O elements, respectively.

To further clarify the catalytic oxidation behaviour of the copper species on the insulating system, the rate of change in the insulating properties at different time points is elaborated in Figure 3a,b. The oil samples containing insulating paper and oil samples containing paper-coated copper are denoted as IP+DBPC and Cu+DBPC, respectively, except for the ones containing DBPC. The dissipation factor, acidity and micro-water increased continuously with the ageing time. The addition of 0.3 wt.% DBPC significantly improved the oxidation resistance of the oil-paper insulation and extended its service life. After the second addition of DBPC, the ageing rates of the dissipation factor, acidity, micro-water and breakdown voltage in the Cu+DBPC group decreased by 28.8%, 43.2%, 52.9% and 46.7%, respectively, compared to the Cu group. Additionally, compared to the IP group, the ageing rates of the dissipation factor, acidity, micro-water and BDV in the IP+DBPC group decreased by 18.4%, 28.3%, 38.3% and 38.6%, respectively, as shown in Figures S5 and S6. During the ageing process, the population of free radicals increases, resulting in an unpaired electron being coupled with a free electron to become a charge carrier. This process contributes to an overall increase in the dissipation factor [42]. The presence of metallic copper tends to increase conductivity, which in turn drops the AC BDV. This is due to the increased conductivity causing the electric field in the transformer oil to become uneven. The breakdown process can be broadly divided into three phases: (1) At low magnetic fields, resistive currents are generated and the increase in the applied electric field reduces the effective barrier at the metal/dielectric interface, which activates the “tunnelling” mechanism, leading to a rapid increase in the injected current in the next phase [43]. (2) Prior to breakdown, in the final stage of a high electric field, the current approaches space charge saturation and the apparent mobility increases. (3) Breakdown occurs when the local electric field strength exceeds the breakdown voltage. High temperatures destroy the molecular chains of the transformer oil, change the properties of the oil and produce ageing products (moisture, Cu species, etc.) and acids, which reduce the insulating properties and cooling effects.

Free radicals are significant chemical reaction intermediates in the ageing process of transformer oil which can induce considerable variations in interfacial tension. The generation of hydroxyl and peroxyl radicals in ageing transformer oil can be indirectly demonstrated by monitoring changes in the interfacial tension and combining the chemical properties of free radicals [44]. As shown in Figure 3c, the rate of decrease in the interfacial tension continued to accelerate. A significant improvement in interfacial tension was demonstrated by the addition of 0.3 wt.% DBPC at 168 h and 336 h. As the most widely used antioxidant in insulating oils, the hydroxyl group in the DBPC molecule provides hydrogen atoms that can react with the reactive free radicals and peroxides in the oil to form stable compounds, thereby interrupting the chain reactions in the oil. This radical-quenching behaviour can improve the antioxidant performance of oil-paper insulation systems to some extent and extend their service life. Fibre polymerisation decreased with increasing ageing time. This is consistent with the above results. The ageing kinetics of insulating paper are contingent upon a number of factors, including high temperature, moisture, acidity, DBPC and Cu species. During ageing, the two-phase structure of cellulose (crystalline and amorphous regions) causes intracellulose compounds to penetrate in two steps. The first step passes through the amorphous region, causing inter-crystalline swelling, leading to the breakage of β-1,4 glycosidic bonds and a decrease in the degree of polymerisation [45]. Simultaneously, cellulose hydrolysis causes the discolouration of the paper and diminishes the degree of polymerisation of the cellulose chains, lowering the strength of the insulating paper [46]. As illustrated in Figure S7, it can be hypothesised that in conditions such as elevated temperatures and the presence of radical species, the cellulose in insulating paper undergoes a sequence of depolymerisation, oxidation and dehydration reactions [44].

It is noteworthy that the fibre degradation rate increased by 47.8% in the Cu+DBPC group compared to the Cu group after the secondary addition of DBPC. Similarly, the rate of fibre degradation in the IP+DBPC group increased by 21.1% compared to the IP group. DBPC, in its phenolic antioxidant form, displays satisfactory stability at room temperature. However, its antioxidant efficacy declines at elevated temperatures, and the resulting ageing products accelerate the deterioration of the oil paper, thereby impairing its insulating properties. The perspective of the structure of matter was used to analyse the mechanism. As shown in Figure 3d, DBPC belongs to the phenolic group, which is weakly acidic due to the interaction between the hydroxyl group and the benzene ring, and thus the phenolic hydroxyl group can exist, to a certain degree, in the presence of ionisation. The higher the content of DBPC added to the oil, the greater the amount of ionised hydrogen ions and the greater the ability to promote the hydrolytic degradation of cellulose, leading to a more intense decrease in the degree of polymerisation in the added DBPC group.

**Figure 4:**
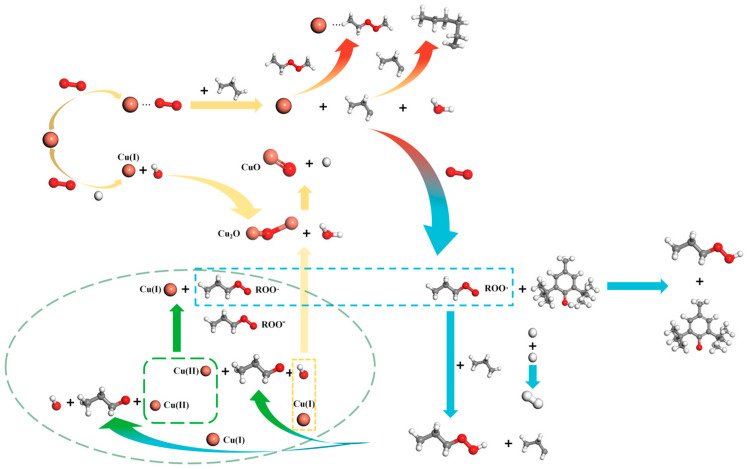
Mechanism of the catalytic oxidation of copper species on oil-paper insulation systems. The white, grey, red and orange spheres represent the H, C, O and Cu elements, respectively.

The catalytic oxidation mechanism for insulating oil paper and Cu species consists mostly of free radical chain reactions. A free radical chain reaction is made up of three steps: chain initiation, chain propagation and chain termination [47]. The catalytic oxidation mechanism of Cu species on oil-paper insulation systems is depicted in Figure 4, and it relies on the generation of peroxides at high temperatures. Peroxide catalysis is mostly based on free radical chain reactions [48]. Under the combined action of high temperature, micro-water and dissolved oxygen, copper acts as an initiator, losing electrons to form the metal ion Cu(I). A portion of Cu(I) species can be stabilised by complexation with a variety of “soft” ligands, which is attributed to the fact that the electronic orbitals of the ligands can hybridise with the d orbitals occupied by Cu(I) [44]. Cu(I) in turn can be further formed into Cu_2_O and CuO for deposition on the fibre surface. With increasing ageing time, the gradual increase in the dissolved copper species in the oil will gradually undergo aggregation, leading to a larger scale. This can be confirmed indirectly by the larger particle size of the copper species on the fibre surface. Simultaneously, copper serves as a catalyst, facilitating the reaction between hydrocarbons and the formation of organometallic compounds and peroxides [49]. The development of free radical chains is prevented, and stable polymer molecules are produced. DBPC can react with reactive free radicals and peroxides produced during petroleum auto-oxidation to form stable compounds that prevent the petroleum oxidation process [50]. This explains the above-mentioned improvement in properties. In addition to thermal initiation, the copper catalyst works as a redox initiator when heated. The metal initiator catalyses the breakdown of peroxides during the chain-branching step, considerably lowering the activation energy of the oxidation process to oxidise the insulating oil at high temperatures. The chain propagation reaction involves adding monomers to the monomer radical to improve the degree of polymerisation. First, the organometallic molecule combines with oxygen, producing alkyl peroxyl radicals. The peroxyalkyl radicals and hydrocarbons are converted to peroxides and new alkyl radicals at high temperatures. The creation of big hydrocarbons during the oxidation process causes a rise in ageing products while limiting the transfer of dissolved oxygen, resulting in chain termination reactions. The conversion of Cu(II) to Cu(I) during the chain termination stage further promotes the generation of Cu_2_O. From a kinetic point of view, according to Le Chatelier’s principle, an increase in the H^+^ inhibits the subsequent reaction to generate CuO, which leads to an increase in the Cu_2_O content detected on the fibre surface. Organometallicperoxides can produce new hydrocarbons, metals and oxygen. The increased activity of free radicals promotes chain termination processes. Free chain development is prevented, and stable polymer molecules are produced.

## 3. Conclusions

In summary, this study reveals the variation in copper scales and the deterioration of insulating properties during the ageing process through a series of accelerated thermal ageing tests. The increase in fibre surface roughness and porosity with an increase in ageing time facilitates the adsorption of more copper onto the fibre surface for deposition and aggregation, which leads to an increase in copper particle size. The copper species dissolved into oil progressively increased as the ageing time progressed. Cu(I) and Cu(II) species were identified as the active sites for inducing intense free radical reactions, resulting in the deterioration of insulating properties. In addition, the time of DBPC incorporation was determined by monitoring the variations in the interfacial tension and taking into account the chemical properties of the free radicals. This free radical-quenching behaviour can improve the antioxidant performance of oil-paper insulation systems to a certain extent and prolong their service life. This work elucidates the catalytic oxidation behaviour of copper-based catalysts on oil-paper insulation systems and implements effective regulation, which provides a useful reference for the improvement of transformer fault warning technology.

## Data Availability

The data presented in this study are available in the article itself.

## Supplementary Materials

The following supporting information can be downloaded at https://www.mdpi.com/article/10.3390/molecules29194690/s1. Materials and Methods; Figure S1: (a–e) SEM images of Cu(504) and corresponding EDS-mapping images; (f) elemental composition; Figure S2: Different ageing time of Cu LMM Auger spectra; Figure S3: XRD pattern of fresh insulating paper; Figure S4: Raman spectra of Cu(504); Figure S5: Insulation properties (micro-water, acidity and dissipation factor) with different ageing times; Figure S6: Insulation properties (interfacial tension, polymerisation degree and breakdown voltage) with different ageing times; Figure S7: Schematic diagram of cellulose degradation; Table S1: Copper species loading on the surface of insulating paper determined by ICP-MS.
