# Supplementary material for: Revealing the Intrinsic Correlation between Cu Scales and Free Radical Chain Reactions in the Regulation of Catalytic Behaviour
Source: Molecules. 2024 Oct 3;29(19):4690. doi: 10.3390/molecules29194690 (PMC11477528; doi:10.3390/molecules29194690)
Supplement: Supplementary file 1 [file molecules-29-04690-s001.zip › molecules-3167103-supplementary.pdf]

Supporting Information

Revealing the intrinsic correlation between Cu scales and free radical chain reaction to regulate the catalytic behaviour

Haifeng Zhang[1], Zilong Zhang[1], Jingyi Yan[1], Siyang Wang[2], Xubin Huang[1], Fangmin Zuo[1], Ao Li[1], Fengkai Gao[3], Haidan Lin[4], and Bolin Wang[1,*]

[1]School of Chemical Engineering, Northeast Electric Power University, Jilin 132012, China;

[2]Department of Chemistry, School of Science, Tianjin Key Laboratory of Molecular Optoelectronic Science, Tianjin University, Tianjin 300072, China;

[3]Science and Technology Industry Division, Northeast Electric Power University, Jilin 132012, China;

[4]Electric Power Research Institute, State Grid Jilin Electric Power Co., Ltd., Changchun 130012, China;

[*]Correspondence: bolinwang@neepu.edu.cn (B.W.)

1 Materials and Methods

1.1 Materials

Karamay insulating oil was purchased from PetroChina Company Limited (Karamay, China). Insulating paper (thickness, 0.07 mm) was purchased from Xuchang Zhongcheng insulating material Co., Ltd. (Xuchang, China). Copper sheet (99.9%, 1 mm) and di-tert-butyl-p-cresol (> 99.5%) were purchased from Macklin Biochemical Co., Ltd. (Shanghai, China). Absolute ethanol (99.7%) was purchased from Aladdin Industrial Corporation (Shanghai, China).

1.2. Experiment setup

The oil samples are vacuum dried at 313 K for 48 h before to ageing, and the measured data from the dried samples serve as the beginning data. The moisture content of the insulating paper was reduced by hoover drying it at 363 K for 48 h. The samples were processed in accordance with reference [1]. Accelerated degradation by heating was conducted on the assumption that remaining life was reduced by one-half every time the temperature rose by 7 K. Since the base temperature of the insulating oil is 333 K, heating at 403 K is equivalent to 1024-fold acceleration ($70/7 = 10$, $2^{10} = 1024$). Based on this concept, the accelerated degradation per day is determined as equivalent to about three-year of aging in the field [2]. All of the test samples were sealed and kept in a vacuum oven at 313 K after ageing. There was nearly little moisture in the oven, therefore the mistake due by external moisture was minimal. Finally, the samples were removed and chilled to room temperature before to testing. IP(168), IP(336), IP(504), Cu(168), Cu(336), and Cu(504) were the names given to the catalysts that were aged for 168 h, 336 h, 504 h. Besides, Oil samples with DBPC and containing paper-coated copper are named Cu+DBPC. Oil samples with DBPC and containing only insulating paper are named IP+DBPC. Both additions of DBPC were at 0.3 wt.%. The ratio of the variation in insulation properties to 168 h is the ageing rate.

1.3. Characterization

XPS was taken on ESCALAB 250Xi with an Al Kα X-ray source (hν = 1486.6 eV) and binding energy referenced to C1s (284.8 eV). XPS was used to analyse the metal

valence evolution of the innermost insulating paper. X-ray diffraction (XRD) patterns were performed on a Bruker D8 Advance diffractometer with Cu-Kα radiation at room temperature. Raman spectrum of Cu species were collected on a Renishaw inVia Raman microscope (inVia Qontor) using 532 nm excitation wavelength. XRD and Raman are used to identify the copper species and crystal structure. Scanning Electron Microscope (SEM) and energy dispersive X-ray (EDS) were taken on SU8100 for the morphology and elemental analyse of the insulating paper. Transmission electron microscopy (TEM) was taken on FEI TalosF200S for the morphology of Cu species.

1.4. Performance evaluation

The micro-water test was used to monitor the degree of moisture in the insulation. Injecting 0.1 μL of pure water with a value of 95-105 ppm at first. Samples are measured by injecting 1 mL of sample at a time, testing three times and take the average value of the samples for the micro-water content. Acidity was used to determine the degree of deterioration of the insulating oil. Firstly, the neutralisation and extraction channels are vented, then pour samples with the same volume into the dry oil cup and put in the rotor of equal size, put the oil cup into the automatic acid value detector for measurement, repeat the above steps and take the average value of 3 measurements as the acid value of the sample. The dimension of the value of the dissipation factor determines the level of degradation and contamination of the transformer oil. Before carrying out the test of dissipation factor, the oil cup was thoroughly cleaned with acetone and deionised water and placed in the oven to dry for 1 h-2 h, then the cup was lubricated twice with samples and the electrodes were assembled. Turn on the thermostat control device, and heat the test oil to the specified temperature of 363 K, then add 40 mL-45 mL of samples during the test. Interfacial tension was a better indicator of oil quality and ageing from the point of view of how much polar material is present than acidity is from the amount of acid (i.e. polar material) present. The automatic interfacial tension tester is calibrated with weights before testing, and followed by pure water calibration. When testing oil samples in the sample cup filled with pure water to the scale line, according to the instrument screen

prompts to add the appropriate amount of oil samples, and adjust the height of the test bench, so that the platinum ring immersed in the liquid under 5 mm, with the platinum ring through the oil-water interface will appear a layer of liquid film, when the liquid film rupture, the measured interfacial tension value is calculated and displayed. The breakdown voltage test cannot tell if the oil includes acids or sludge, but it may indirectly tell whether it has water, contaminants, and conductive particles. Oil breakdown tester was used to measure ac breakdown voltages, using brass spherically capped electrodes. There was a 30 minutes settling time between breakdowns. Either six or twelve breakdowns were performed per set depending on the value.

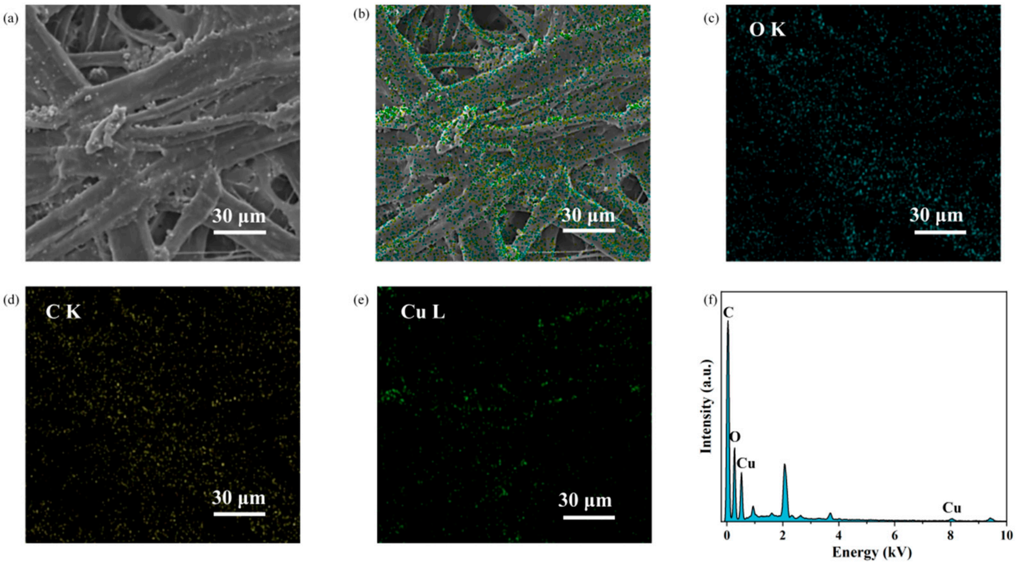

Figure S1 (a)-(e) SEM images of Cu(504) and correspnding EDS-mapping images; (f) Elemental composition.

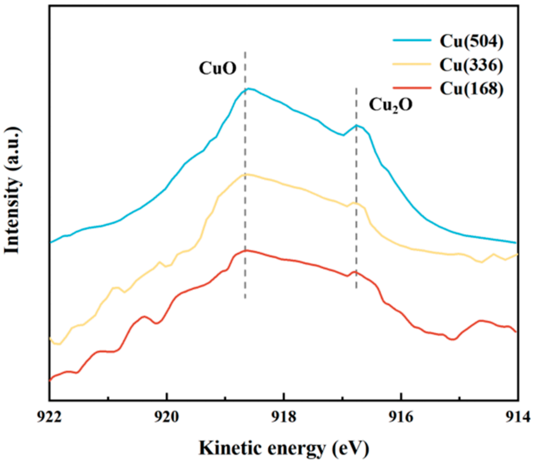

Figure S2 Different ageing time of Cu LMM Auger spectra

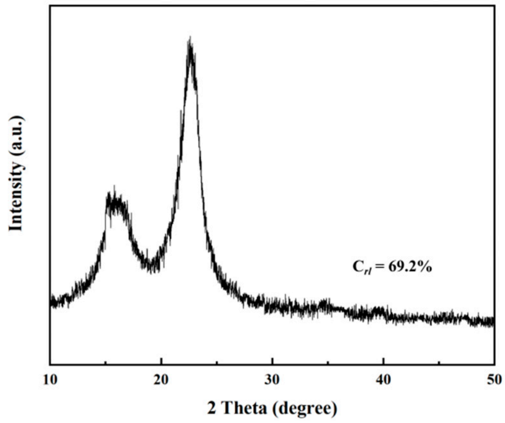

Figure S3 XRD pattern of fresh insulating paper.

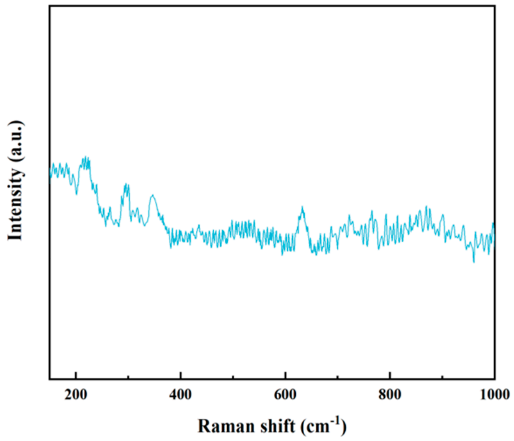

Figure S4 Raman spectra of Cu(504)

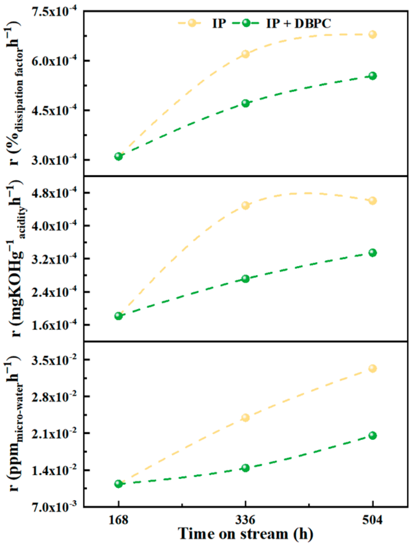

Figure S5 Insulation properties (micro-water, acidity, dissipation factor) with different ageing times.

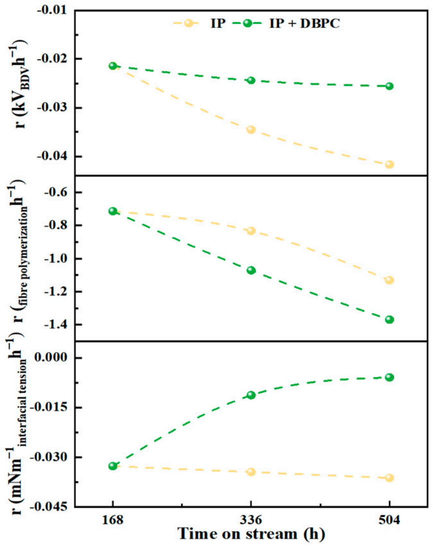

Figure S6 Insulation properties (interfacial tension, polymerization degree, breakdown voltage) with different ageing times.

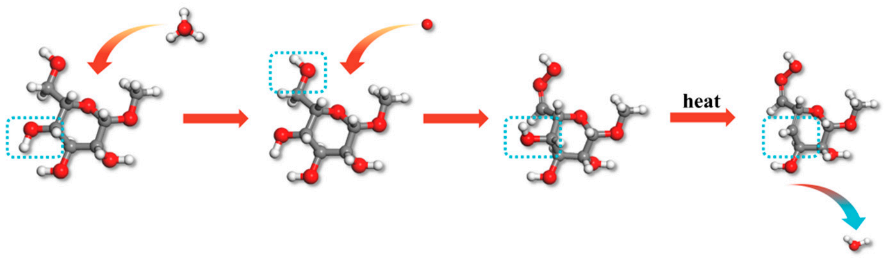

Figure S7. Schematic diagram of cellulose degradation.

Table S1. Copper species loading on the surface of insulating paper determinted by ICP-MS

Catalyst	Cu loading (wt.%)
IP	0.00
Cu(168)	1.62
Cu(336)	5.24
Cu(504)	7.13

Reference

1. X.F. Hu, H.X. Cong, Y.X. Wang, X. Zhang, Y.L. Du, Q.M. Li, Z.B. Yu, Experimental research on deterioration effect of transition metals on natural ester, IEEE Trans. Dielectr. Electr. Insul., 31 (2023) 477-484.
2. J. Wada, G. Ueta, S. Okabe, T. Amimoto, Inhibition technique of transformer insulating oil degradation-evaluation of the effectiveness of oxidation degradation inhibitors, IEEE Trans. Dielectr. Electr. Insul., 20 (2013) 1641-1648.
